# Supplementary material for: Vasoprotective Functions of High-Density Lipoproteins Relevant to Alzheimer’s Disease Are Partially Conserved in Apolipoprotein B-Depleted Plasma
Source: Int J Mol Sci. 2019 Jan 22;20(3):462. doi: 10.3390/ijms20030462 (PMC6387156; doi:10.3390/ijms20030462)
Supplement: Supplementary file 1 [file ijms-20-00462-s001.pdf]

Supplemental Figure 1

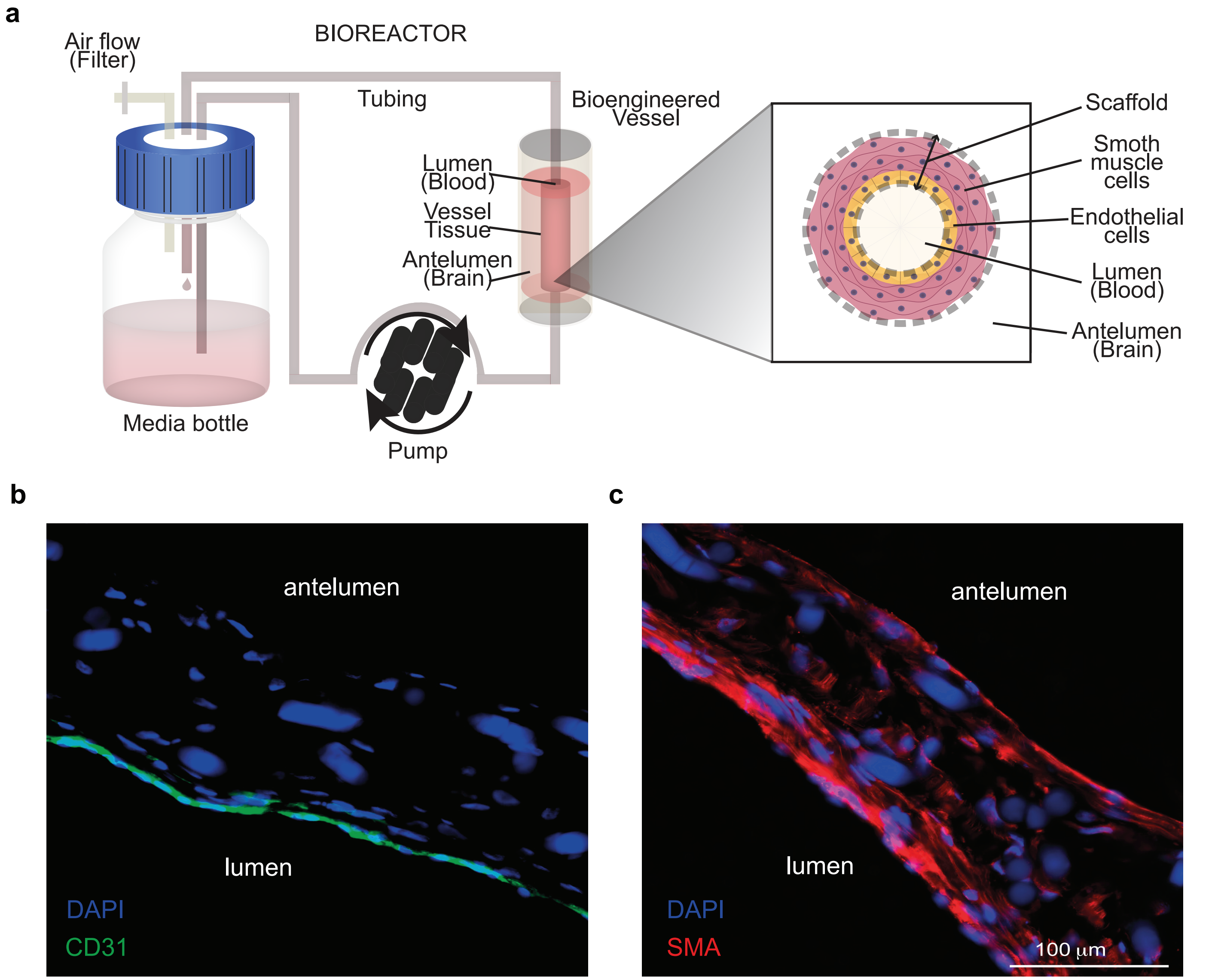

Supplemental Figure 1: ***Bioengineering 3D human arteries.***
(**a**) 3D bioengineered human vessels were subjected to abluminal Aβ42 treatment with or without luminal HDL treatment. Histological characterization of the vessels shows (**b**) a monolayer of endothelial cells on the luminal aspect visualized with CD31 and (**c**) multiple layers of smooth muscle cells throughout the vessel visualized with smooth muscle actin (SMA). CD31: cluster of differentiation 31, SMA: smooth muscle actin, DAPI: 4′,6-di-amidino-2-phenylindole.

Supplemental Figure 2

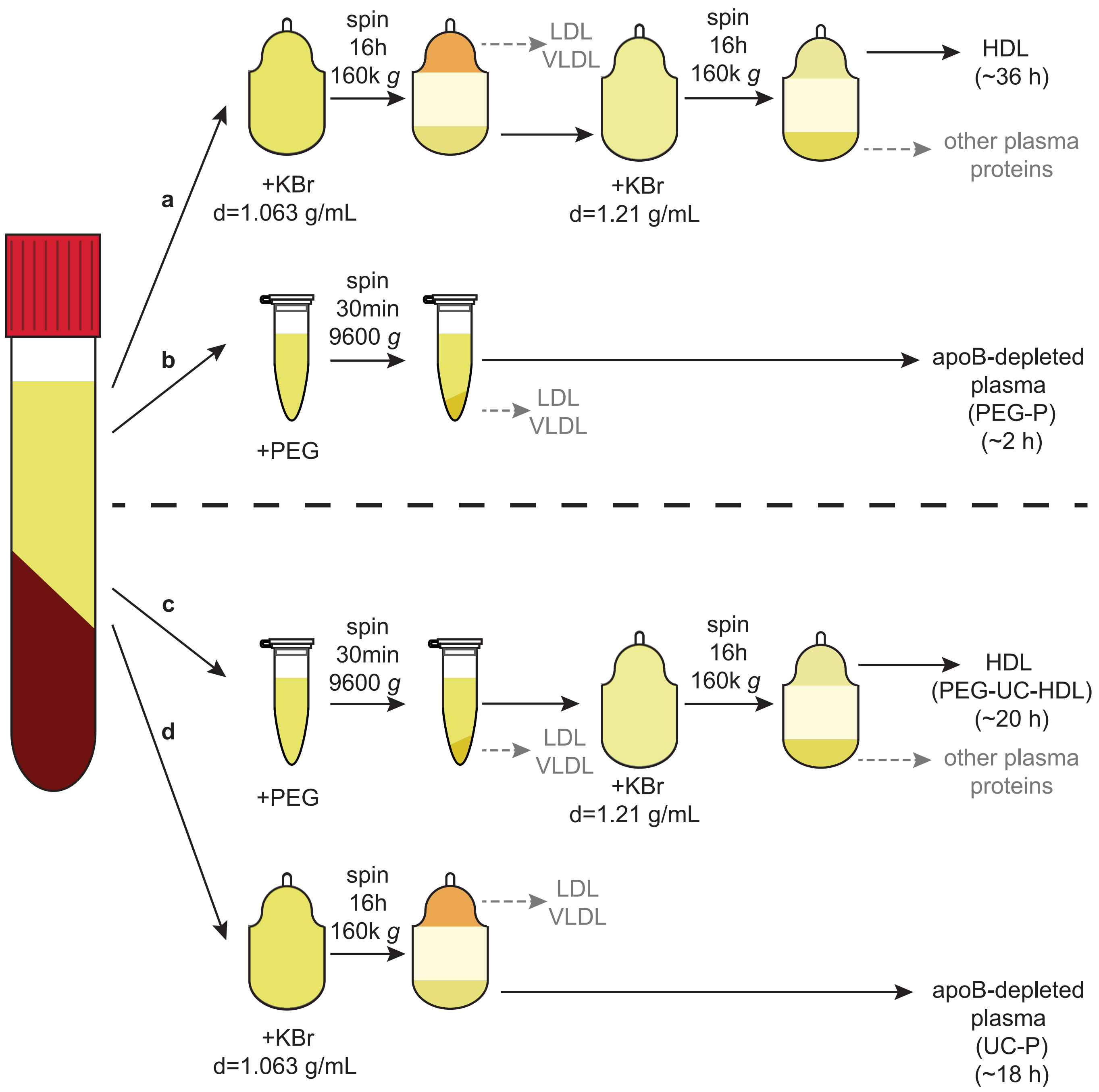

Supplemental Figure 2: ***HDL isolation and depletion of apoB-containing lipoproteins from human plasma.***
HDL or apoB-depleted plasma was prepared from young, healthy donor plasma by (**a**) sequential density gradient ultracentrifugation (HDL) using KBr, (**b**) polyethylene glycol (PEG-P) precipitation of non-HDL lipoproteins, (**c**) a combination of the two methods (PEG-UC), or (**d**) a single UC spin (UC-P). KBr: potassium bromide, LDL: low-density lipoprotein, VLDL: very low-density lipoprotein, HDL: high-density lipoprotein.

Supplemental Figure 3

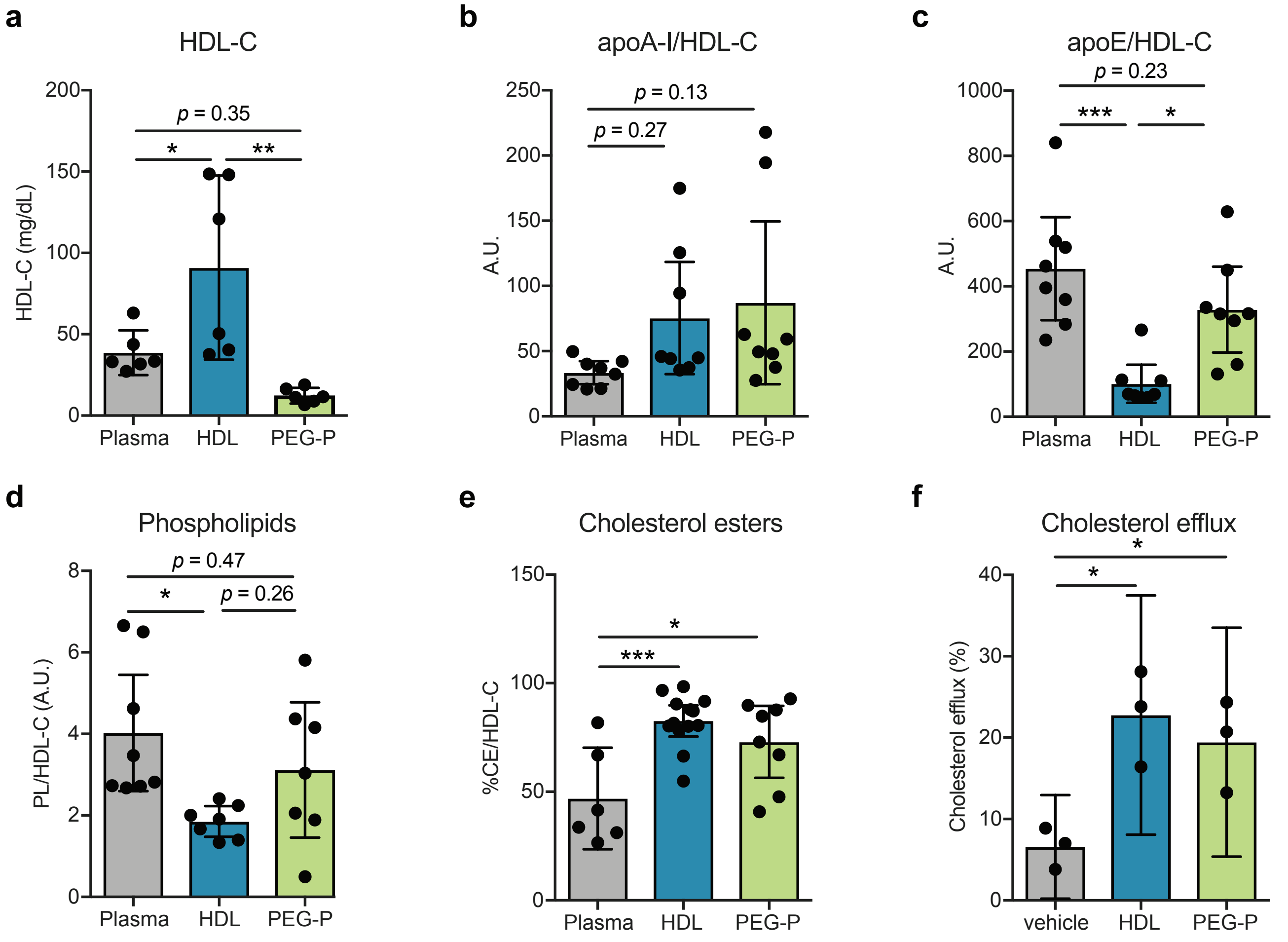

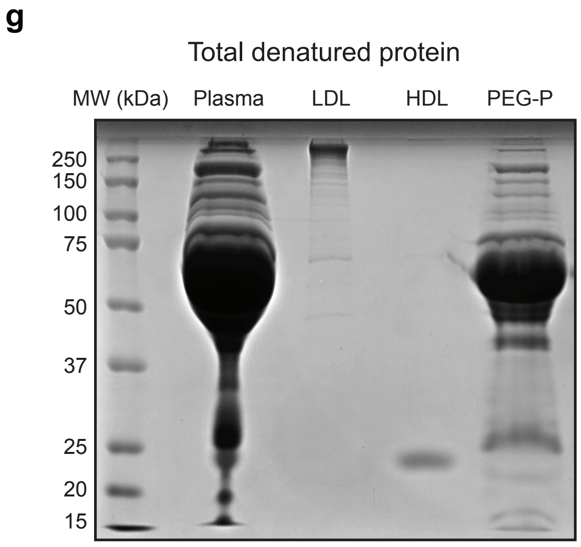

Supplemental Figure 3: *Composition and cholesterol efflux function of HDL and apoB-depleted plasma.* The levels of (**a**) HDL-C, (**b**) apoA-I and (**c**) apoE, (**d**) phospholipid, and (**e**) cholesterol ester levels were measured in unfractionated plasma, HDL isolated from plasma by ultracentrifugation, and apoB-depleted plasma produced by PEG precipitation and normalized to HDL-C concentration. (**f**) Cholesterol efflux capacity was measured using RAW 264.7 macrophages loaded with ^{3}H-cholesterol. (**g**) Total protein was observed in HDL using SDS-PAGE denaturing gels stained with Coomassie Brilliant Blue. Scatter plots represent independent experiments with mean ±95% confidence interval. *p<0.05, **p<0.01, ***p<0.001, or exact p-values by one-way analysis of variance (ANOVA) with Tukey's multiple comparisons test. MW: molecular weight, d: diameter, kDa: kilodalton, LDL: low-density lipoprotein, HDL: high-density lipoproteins isolated by sequential density gradient ultracentrifugation, PEG-P: apoB-depleted plasma by polyethylene glycol precipitation, A.U.: arbitrary units, SDS-PAGE: sodium dodecyl sulphate polyacrylamide gel electrophoresis.
